# Supplementary material for: Screening autism-associated environmental factors in differentiating human neural progenitors with fractional factorial design-based transcriptomics
Source: Sci Rep. 2023 Jun 29;13:10519. doi: 10.1038/s41598-023-37488-0 (PMC10310850; doi:10.1038/s41598-023-37488-0)
Supplement: Supplementary file 1 — Supplementary Information 1. [file 41598_2023_37488_MOESM1_ESM.pdf]

Supplementary Information

Screening autism-associated environmental factors in differentiating human neural progenitors with fractional factorial design-based transcriptomics

Abishek Arora, Martin Becker, Cátia Marques, Marika Oksanen, Danyang Li, Francesca Mastropasqua, Michelle Evelyn Watts, Manish Arora, Anna Falk, Carsten Oliver Daub, Ingela Lanekoff and Kristiina Tammimies*

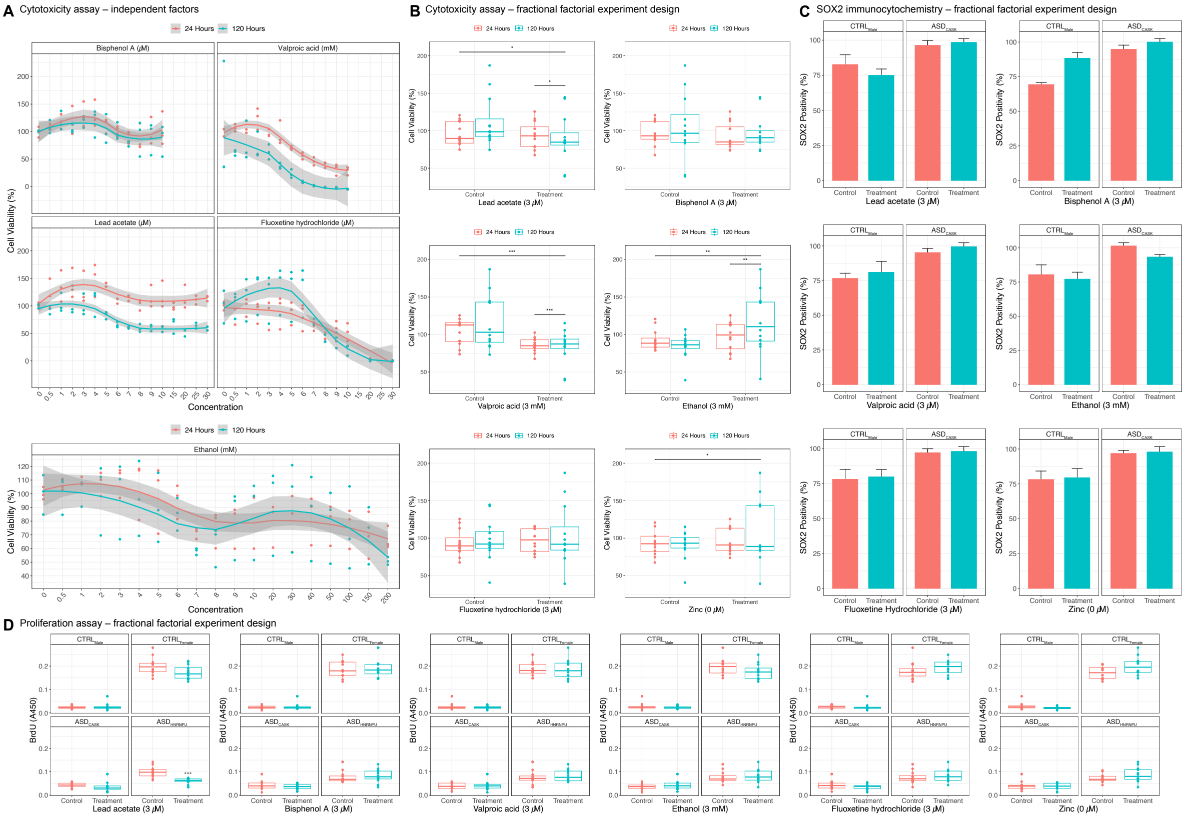

Figure S1. Cellular effects of environmental factors on differentiating neural progenitors. (**A**) Estimation of cell viability following exposure of lead (Pb), valproic acid (VPA), bisphenol A (BPA), fluoxetine (FH) and ethanol (EtOH) for 24 and 120 hours (5 days) on undirected differentiation of CTRL$_{Male}$ using the MTS cytotoxicity assay. (**B**) Estimation of cell viability for selected concentrations (from A) of Pb, VPA, BPA, EtOh, FH, and zinc deficiency (Zn-) for 24 and 120 hours (5 days) on undirected differentiation of CTRL$_{Male}$ using the fractional factorial experiment design (FFED) and MTS cytotoxicity assay (Tukey post hoc p *<0.05, **<0.01, ***<0.001). (**C**) SOX2 positivity following exposures of selected concentrations (from A) of Pb, VPA, BPA, EtOh, FH and Zn- in FFED for 120 hours (5 days) on undirected differentiation of CTRL$_{Male}$ and ASD$_{CASK}$. (**D**) Changes in cellular proliferation from exposures of selected concentrations (from A) of Pb, VPA, BPA, EtOH, FH, and Zn- in FFED at 120 hours (5 days) on undirected differentiation of CTRL$_{Male}$, CTRL$_{Female}$, ASD$_{CASK}$ and ASD$_{HNRNPU}$ using the BrdU assay (Tukey post hoc p ***<0.001).

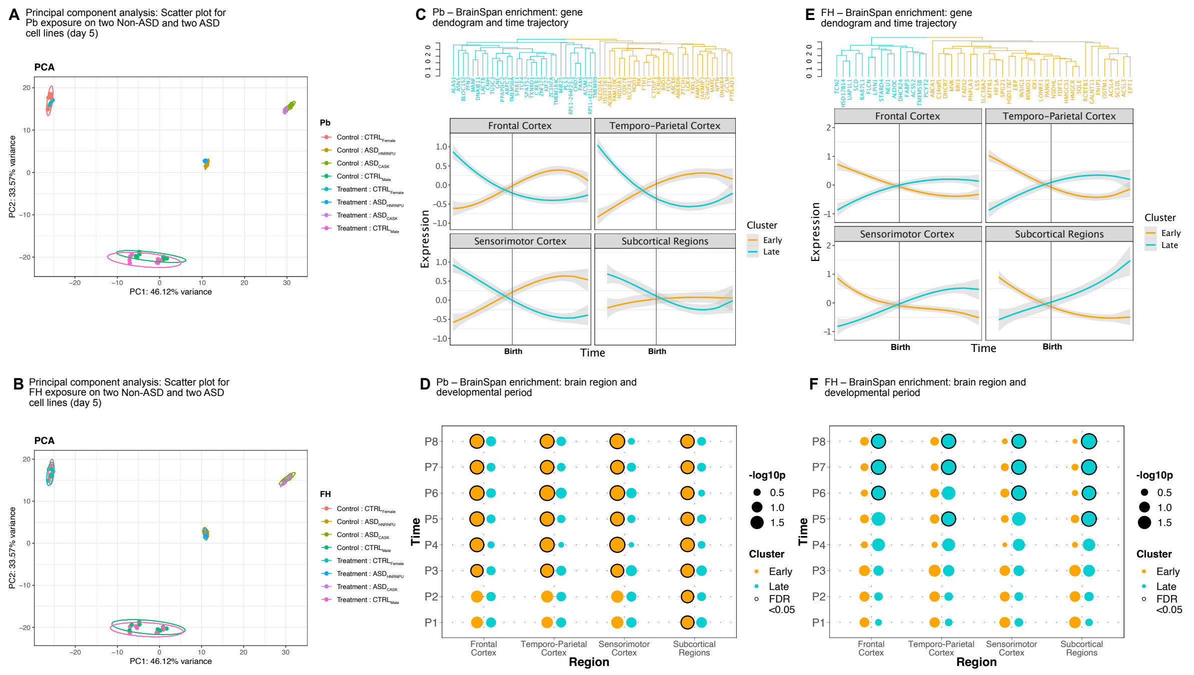

Figure S2. Differential expression and gene enrichment for lead (Pb) and fluoxetine (FH). (**A**) Principal component analysis (PCA) scatter plot of non-ASD and ASD cell lines exposed to Pb for 5 days. (**B**) PCA scatter plot of non-ASD and ASD cell lines exposed to FH for 5 days. (**C**) Cluster dendogram and developmental period trajectory of significant differentially expressed genes (DEGs) following Pb exposure for 5 days, in the Brainspan dataset. (**D**) Enrichment dot plot of significant DEGs following Pb exposure for 5 days, in the Brainspan dataset across four brain regions (Frontal Cortex, Temporo-parietal Cortex, Sensorimotor Cortex, and Subcortical Regions) for developmental periods (P1: Early foetal<=12 weeks, P2: Early mid-foetal 13-18 weeks, P3: Late mid-foetal 19-24 weeks, P4: Late foetal 25-38 weeks, P5: Infancy 18 months, P6: Childhood 19 months-11 years, P7: Adolescence 12-19 years, P8: Adulthood 20-60+ years). (**E**) Cluster dendogram and developmental period trajectory of significant DEGs following FH exposure for 5 days, in the Brainspan dataset. (**F**) Enrichment dot plot of significant DEGs following FH exposure for 5 days, in the Brainspan dataset across four brain regions (Frontal Cortex, Temporo-parietal Cortex, Sensorimotor Cortex, and Subcortical Regions) for developmental periods (P1: Early foetal<=12 weeks, P2: Early mid-foetal 13-18 weeks, P3: Late mid-foetal 19-24 weeks, P4: Late foetal 25-38 weeks, P5: Infancy 18 months, P6: Childhood 19 months-11 years, P7: Adolescence 12-19 years, P8: Adulthood 20-60+ years).

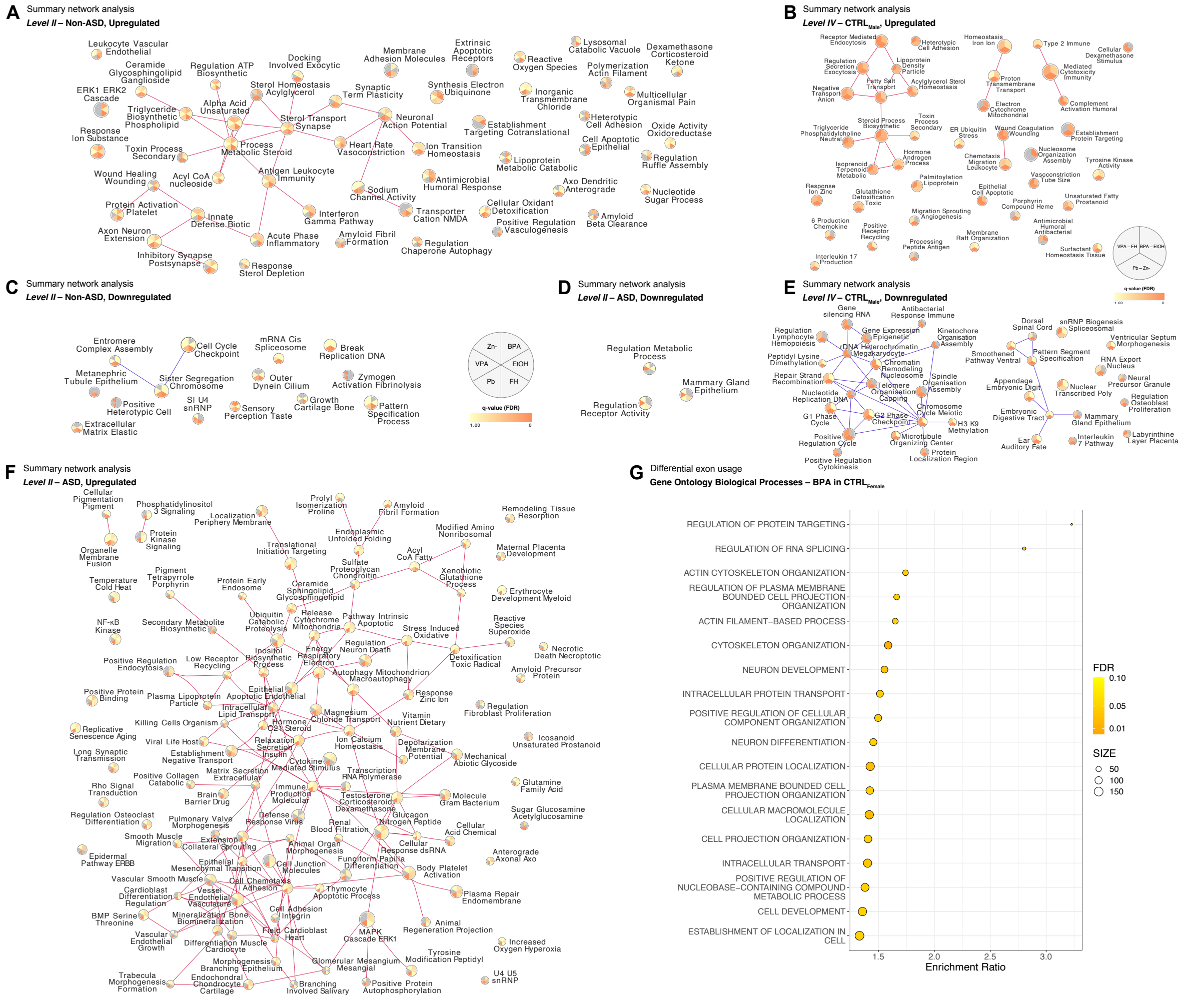

Figure S3. Summary networks and differential exon usage (DEU) of selected environmental factors in differentiating neural progenitors. (**A**) Upregulated clusters in the non-ASD cell lines (*Level II* analysis). (**B**) Upregulated clusters for two-way interactions in $\text{CTRL}_{\text{Male}}$ (*Level IV* analysis). (**C**) Downregulated clusters in the non-ASD cell lines (*Level II* analysis). (**D**) Downregulated clusters in the ASD cell lines (*Level II* analysis). (**E**) Downregulated clusters for two-way interactions in $\text{CTRL}_{\text{Male}}$ (*Level IV* analysis). (**F**) Upregulated clusters in the ASD cell lines (*Level II* analysis). (**G**) Gene ontology biological processes (GOBP) enrichment terms of significant DEU genes (adj. $p<0.05$) following bisphenol A (BPA) exposure in $\text{CTRL}_{\text{Female}}$ (*Level III* analysis).

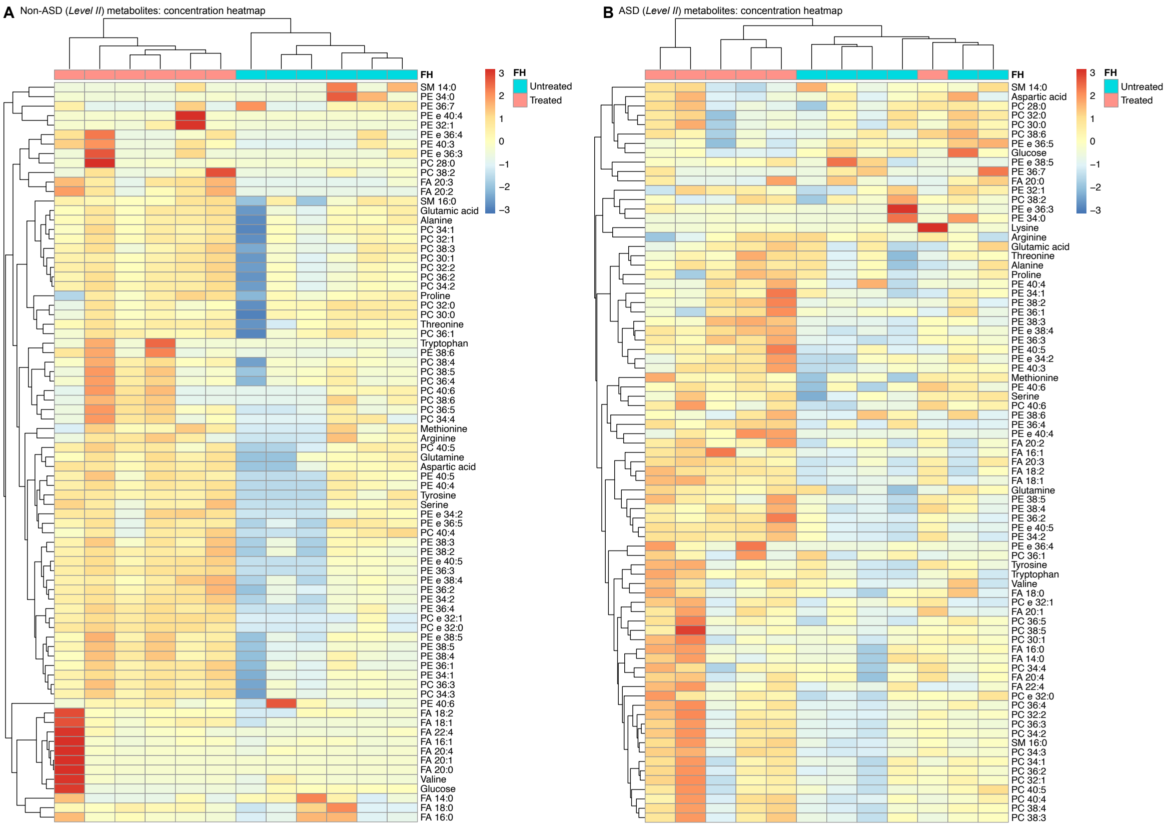

Figure S4. Mass spectrometry-based metabolomics of fluoxetine (FH) exposure. (**A**) Concentration heatmap of selected metabolites following FH exposure for 5 days in the non-ASD cell lines. (**B**) Concentration heatmap of selected metabolites following FH exposure for 5 days in the ASD cell lines.
